# Maintenance of Remission with Partial Enteral Nutrition Therapy in Pediatric Crohn's Disease: A Retrospective Study

**DOI:** 10.1155/2017/5873158

**Published:** 2017-05-08

**Authors:** Jacqueline M. Schulman, Liat Pritzker, Ron Shaoul

**Affiliations:** ^1^The Ruth and Bruce Rappaport Faculty of Medicine, Technion-Israel Institute of Technology, 3525433 Haifa, Israel; ^2^Pediatric Gastroenterology & Nutrition Unit, Ruth Children's Hospital, Rambam Medical Center, 3109601 Haifa, Israel

## Abstract

**Background:**

Partial enteral nutrition (PEN) may be helpful for the maintenance of remission in pediatric Crohn's disease patients.

**Aims:**

To evaluate the efficacy of PEN treatment for preventing clinical relapse.

**Methods:**

We retrospectively assessed 42 pediatric Crohn's disease patients who entered clinical remission on 4–12 weeks of exclusive enteral nutrition (EEN) and were maintained on PEN as a supplementary diet. We evaluated the efficacy of the treatment at different time points using the weighted Pediatric Crohn Disease Activity Index (wPCDAI), Physician Global Assessment, laboratory parameters, and growth of each patient. Additionally, we assessed the use of concomitant medications.

**Results:**

The median length of remission with PEN was 6 (0–36) months. Patients' remission was maintained on PEN without concomitant medications for a median time of zero months (0–16). The mean body mass index in the PEN group increased from 18.1 to 18.8 after six months of PEN. The median wPCDAI decreased from 30 at diagnosis to 5.0 after EEN and increased to 7.5 after three months of PEN. Overall, the median wPCDAI decreased by 26.2.

**Conclusions:**

PEN treatment was partially effective in maintaining remission and was able to increase BMI and lower wPCDAI. Most patients required concomitant medication after PEN initiation.

## 1. Introduction

Crohn's disease (CD) is a chronic disease that currently lacks a cure. Aside from the widespread prevalence of CD in adult patients, there is a rising incidence and prevalence in the pediatric population [1–3]. Treatment of CD patients involves a multidisciplinary approach that aims to induce and maintain clinical, laboratory, and mucosal remission and to ultimately prevent relapses. Current treatment of pediatric CD includes exclusive enteral nutrition (EEN), corticosteroids, immunomodulators, antibiotics, and biological agents together with psychological and psychiatric support.

Pediatric patients with CD are at constant risk of malnutrition, and, thus, proper nutrition is a vital aspect of the treatment of the disease. The liquid diet used in EEN was developed as an alternative to corticosteroid therapy for induction of remission and is gaining worldwide popularity in the medical community. Due to its excellent safety profile and its equipotential to corticosteroids in inducing remission, EEN is now considered a first-line agent to induce remission in children with active CD in many parts of the world [4]. EEN is associated with significantly fewer adverse effects, compared with corticosteroids. Using EEN instead of corticosteroids in pediatric patients has been shown to promote better linear growth, improve bone health, and achieve better mucosal healing [5–7].

As a supplement to their regular diet, partial enteral nutrition (PEN) has proven to be beneficial in the maintenance of remission in adults, especially after EEN treatment [8–13]. Long-term PEN has been shown to be an effective means of decreasing clinical relapse rates and in suppressing endoscopic disease activity [14]. A retrospective study by Wilschanski et al. that investigated the effects of PEN in the pediatric population showed that PEN treatment of children with CD is associated with prolonged remission and better linear growth after treatment with EEN. The patients were allowed a normal daytime diet and received the PEN diet through a nasogastric feeding tube at night [15]. Although several studies suggest that PEN may be helpful for the maintenance of remission in the pediatric population [15, 16], data on the long-term usage of PEN for remission maintenance in pediatric CD patients is still lacking.

We performed a retrospective study to investigate the efficacy of PEN treatment by analyzing the weighted Pediatric Crohn Disease Activity Index (wPCDAI), the Physician Global Assessment (PGA), and laboratory parameters, such as C-reactive protein (CRP) and albumin, as well as the growth of each patient. In particular, we aimed to gain more insight into PEN as a means of preventing clinical relapse, with the goal of improving the treatment for more effective maintenance of remission.

## 2. Materials and Methods

In our study, we retrospectively assessed pediatric CD patients treated in our clinic. The local ethics board at Rambam Medical Center approved the study and waived the requirement for informed consent. We reviewed the clinical records of 48 children aged 1–20 years diagnosed with CD during the period from January 2001 to June 2013. The patients entered clinical remission on 4–12 weeks of EEN and were subsequently maintained on PEN (50% of total calories as polymeric diet, usually Modulen® IBD) as a supplementary diet. We evaluated the efficacy of the treatment at different time points using the wPCDAI, PGA, laboratory parameters (CRP and albumin), and the growth of each patient in terms of weight, height, and body mass index (BMI). Also, we assessed the use of concomitant medication, such as corticosteroids, thiopurines, anti-TNF agents, methotrexate, and antibiotics in patients maintained on PEN. Children who were not treated with EEN followed by PEN, or for whom we lacked data, were excluded from the study. Six of the 48 patients were excluded from analysis, due to a lack of data, leaving a total of 42 patients to be included in the analysis. Children with CD who refused EN served as the control group, which consisted of 45 patients aged 1–18 years diagnosed with CD between August 2004 and June 2013. Patients in this group were evaluated at different time points using the wPCDAI, PGA, laboratory parameters (CRP and albumin), and the growth of each patient in terms of weight, height, and body mass index (BMI).

Anatomic location and behavior of the disease were evaluated using the Paris classification [17]. The wPCDAI [18] and PGA were used to assess the clinical activity of the disease, with the remission and relapses determined by the judgment of the physician.

Three types of EN were used: Modulen (Nestlé Health Science, Vevey, Switzerland) (90.7%), Ensure® (Abbott Nutrition, Columbus, Ohio) (7.0%), and PediaSure® (Abbott Nutrition, Columbus, Ohio) (2.3%). Further details on the different formulas can be found in the supporting information (Supplementary Table  1, in Supplementary Material available online at https://doi.org/10.1155/2017/5873158). The route of administration was oral, but if not tolerated, nasogastric feeding was used instead.

The patients were followed up regularly, with clinic appointments about once every 4–8 weeks.

Univariate analysis was done with Chi-square or Fisher's exact test for categorical data. Kolmogorov–Smirnov test was used for measuring normal distribution of the quantitative parameters. Based on the result obtained, we chose whether to use a nonparametric test (Mann–Whitney* U* test) or parametric test (*t*-test) for the assessment of differences between groups. A multivariate linear regression model was used for the prediction of length of remission with several independent parameters. A *P* value < 0.05 was considered to be statistically significant in a two-sided test. Statistical analyses were performed with the IBM® SPSS® Statistics software version 21.

## 3. Results and Discussion

### 3.1. Results

A total of 42 patients received EEN therapy to induce remission, followed by PEN therapy to maintain remission. Out of these, 30 (71%) were males, and the median age at diagnosis of the patients was 11.5 years (25–75% = 9.2–13.7 years). Patient characteristics at the time of diagnosis are illustrated in Table 1. The control group, which did not receive EN, consisted of 45 patients in total, of whom 31 (69%) were males. The median age at diagnosis in the control group was 14.7 years (25–75% = 12.7–16.3 years).

The median time between diagnosis of the disease and initiation of EEN was 2.0 months (range, 0–112 months). Thirty-seven patients (88%) received EN orally, whereas four patients (10%) required nasogastric feeding, and one patient received feeding via gastrostomy (2%).

Patients on PEN were followed up for a median time of 40 months (25–75% = 25.5–79 months) in the PEN group, while patients in the control group were followed for a median time of 63 months (25–75% = 52–87 months, *P* = 0.005). The resulting median length of remission with PEN was 6.0 months, and the longest remission period was 36 months. The median length of remission in the control group was 6.0 months, and the longest remission period achieved was 45 months (*P* = 0.96). Additional medications, including corticosteroids, thiopurines, anti-TNF agents, methotrexate, and antibiotics, were added to patients according to pediatric gastroenterologist discretion. Of the 42 patients on PEN, 26 (62%) received corticosteroids, 35 (83%) received thiopurines, 14 (33%) received anti-TNF agents, 7 (17%) received methotrexate, and 19 (45%) were given antibiotics at some point. Among the control subjects, 35 (78%) received corticosteroids, 30 (67%) received thiopurines, 10 (22%) received anti-TNF agents, 2 (4%) received methotrexate, and 19 (42%) were given antibiotics at some point. In more than 50% of the patients, remission could not be maintained with PEN alone without the addition of concomitant medications. Those who received concomitant medication in parallel to PEN were analyzed separately from those who received concomitant medication later. Although the parallel group had a longer remission (10.4 versus 8.6 months), the difference was not statistically significant. A summary of the remission rates and use of concomitant medication is presented in Table 2. In the multivariate analysis, there were no significant predictors for length of remission.

Patients were followed up in terms of various laboratory parameters. In the PEN group, albumin and CRP were recorded before EEN initiation, at the end of EEN therapy, and after three months of PEN treatment. With EEN therapy, there was an increase in the mean albumin level and a decrease in the median CRP, followed by a decrease in albumin and an increase in CRP with PEN. In the control group, albumin and CRP were recorded at diagnosis, after eight weeks, and after five months. The mean albumin level increased after eight weeks and then remained the same after an additional three months, whereas the median CRP decreased after eight weeks and then further decreased after another three months. The laboratory parameters and their changes over time are listed in Table 3.

At each appointment, the height and weight of the patients were measured, and the BMI was calculated. The measurements were recorded and compared before EEN, after EEN, and after PEN in the PEN group. Similarly, in the control group, the measurements were recorded at diagnosis, eight weeks after diagnosis, and eight months after diagnosis. In the PEN group, the mean weight increased with EEN and then further increased with six months of PEN. The mean height did not change with EEN but did increase with six months of PEN. The mean BMI was higher after six months of PEN than before or after EEN. Among the control subjects, the mean weight increased eight weeks after diagnosis and then further increased after an additional six months. The mean height decreased eight weeks after diagnosis but then increased after another six months. The mean BMI was higher after eight months than at diagnosis or at eight weeks after diagnosis. The changes in mean weight, height, and BMI are illustrated in Table 4.

The wPCDAI was determined at the following time intervals in the PEN group: before EEN, after EEN, after three months of PEN, as well as after one, two, and three years of PEN. The results are shown in Table 4. The median wPCDAI decreased from 30.0 at diagnosis to 5.0 after completion of EEN and then increased to 7.5 after three months of PEN. The lowest median wPCDAI of 3.8 was achieved after completion of one year of PEN. In the control group, the wPCDAI was determined at diagnosis and then at eight weeks and five months after diagnosis, as well as at one, two, and three years after diagnosis. The median wPCDAI decreased from 30.0 at diagnosis to 10.0 after eight weeks and then increased to 11.3 after an additional three months. The lowest median wPCDAI was 10.0, which was achieved after eight weeks and at one year and two years after diagnosis. Overall, the median wPCDAI decreased by a total of 26.2 in the PEN group, as compared to a decrease of 20.0 in the control group. The changes in wPCDAI over time are summarized in Table 4.

## 4. Discussion

Currently, the main medications used for maintaining remission in pediatric CD are methotrexate and azathioprine. Both are associated with significant adverse effects. Methotrexate is associated with long-term liver injury, bone marrow depression, and occasionally significant nausea and vomiting around administration. Azathioprine is associated with pancreatitis, bone marrow depression, and long-term increased incidence of lymphoma. In addition, the one-year maintenance of remission is around 30% and 60% for methotrexate and azathioprine, respectively [4]. Therefore, we should aim for a safer and more efficient maintenance regimen, especially in children. PEN, after achievement of remission with EEN, seems a promising alternative. We have practiced this approach for the last several years. This retrospective study aimed to investigate the efficacy of PEN treatment in the maintenance of remission in the pediatric CD population. The total decrease in the mean wPCDAI was greater in the PEN group than in the control group, as was the total increase in BMI between the time of diagnosis and eight months after diagnosis. Laboratory parameters, such as albumin and CRP, also showed better improvement in the PEN group than in the control group. Although PEN was able to maintain short remission in patients initially treated with EEN, more than 50% of the patients required concomitant medications within two weeks of PEN initiation to maintain remission. Most patients required concomitant medication at some point after PEN initiation.

The use of PEN as a treatment for CD has been supported in numerous studies conducted by Yamamoto et al. A 2005 study confirmed that enteral nutrition can induce remission and produce mucosal healing of the intestines [19]. This study was followed by one in which the researchers investigated long-term EN and its effect on clinical and endoscopic disease activities, as well as mucosal tissue cytokines in quiescent CD patients [20]. Cytokines are thought to be important in the modulation of intestinal immune function. In particular, interleukin-1*β* (IL-1*β*), IL-6, and tumor necrosis factor-*α* (TNF-*α*) are thought to play a major role in the aggravation of intestinal inflammation in CD. In this 2007 prospective study, 40 CD patients in remission were divided into two groups. One group, the EN group, received the liquid diet as an infusion at night and a low-fat normal diet during the day, while the non-EN group did not receive the treatment and were not restricted in their diet. The two groups were followed for one year, with mucosal biopsies and ileocolonoscopy performed at entry, 6 months, and 12 months. They found that 5 patients (25%) of the EN group and 13 patients (65%) in the non-EN group relapsed (*P* = 0.03), and the non-EN group had significantly higher endoscopic inflammation (EI) scores at both 6 and 12 months (*P* = 0.04). In addition, while the EN group did not show a noteworthy increase in interleukin-1*β* (IL-1*β*), IL-6, and tumor necrosis factor-*α* (TNF-*α*) cytokine levels in the mucosal tissue, the non-EN group experienced a significant rise in these cytokines with time (IL-1*β*, *P* = 0.02; IL-6, *P* = 0.002; TNF- *α*, *P* = 0.001). These results led the researchers to conclude that long-term EN is effective in decreasing clinical relapse rates and in suppressing endoscopic disease activity, as well as mucosal cytokine levels in patients with quiescent CD.

In a comprehensive review, Yamamoto et al. evaluated the effect of EN on the maintenance of remission in CD patients who had achieved medically or surgically induced remission [14]. EN was associated with significantly higher remission rate and suppressive effects on endoscopic disease activity. Also, the efficacy of EN was shown to be dose-dependent, with higher amounts of enteral formula associated with higher remission rates.

While the available data suggests that EN is effective in maintaining remission in CD patients, the evidence level is not high. Its mechanism of action is not fully understood and remission is often short-lived, with many patients experiencing relapse and requiring upgrading of the therapy or surgery [21]. Furthermore, a significant portion of the studies have been conducted in Japan, as well as in adult populations, and their results may not always apply to pediatric patients in Western communities. A 2006 study by Johnson et al. comparing PEN with EEN suggested that PEN does not suppress inflammation and that EEN is more effective in inducing remission in children with active CD [22]. It is often, however, difficult for children to adhere to EEN in the long-term, resulting eventually in low compliance rates. Thus, the use of PEN for the maintenance of remission after induction with EEN becomes an important consideration.

Several recent studies have supported the findings of Wilschanski et al. that showed that long-term PEN treatment is an effective means of maintaining remission in pediatric CD patients [15]. Konno et al. concluded that EN therapy leads to a reduction in the rate of disease complications, such as intestinal surgeries. In contrast to our study, however, the authors found that EN treatment was capable of maintaining remission in pediatric CD patients for long periods of time without the concomitant use of corticosteroids and immunosuppressive medications [23]. In addition, Kang et al. showed in a prospective study that short-term PEN was effective in improving nutritional status in pediatric patients with severe CD [24]. The results of our study further support these findings. A potential approach to further improve the maintenance of remission in pediatric CD patients on PEN is to combine PEN with a carefully restricted diet. In a recent study, Sigall-Boneh et al. treated a group of children and young adults suffering from CD with a 6-week exclusion diet consisting of 50% PEN and whole foods, with restricted exposure to other dietary products. The dietary intervention resulted in induction of clinical remission and reduction of inflammatory markers in patients suffering from mild-to-moderate disease [25].

Several limitations of our study need to be acknowledged. First of all, the retrospective design of the study may increase the risk of selection bias. Furthermore, the number of EEN weeks needed to induce remission in the PEN group ranged from 4 to 12 and thus was not standardized. Three types of EN formula, Modulen, Ensure, and PediaSure, were used, introducing another degree of variability in the intervention. Nevertheless, almost all (90.7%) of the patients in the PEN group were treated with Modulen. We also acknowledge some key differences in the characteristics of the PEN group and control group. Namely, the median age of the control group was significantly older than that of the PEN group. There was also more colonic disease in the control group, although this was not statistically significant. Finally, the relatively small number of patients included in our investigation may introduce some bias and decrease the power of the study. Our study did include a control group, and therefore we were able to compare pediatric CD patients who were treated with long-term PEN with those who did not receive the treatment. It is important to note, however, that the control group did not receive a course of EEN prior to being maintained on their regular diet. This may cause a confounding effect on the results of the study. Additional studies that compare a study group receiving PEN following an EEN induction with a control group receiving a regular diet after EEN would be of significant value. Moreover, randomized clinical trials that compare treatment with PEN to treatment with medications only, such as corticosteroids and immunomodulators, are needed to identify the optimal management.

## 5. Conclusions

We conclude that PEN treatment was partially effective in maintaining remission in patients who were initially treated with EEN. Also, EN therapy was able to increase patients' BMI and lower their wPCDAI, further supporting its use over corticosteroids in pediatric patients. Most patients required concomitant medication at some point after PEN initiation. To better assess the efficacy of PEN for maintaining remission in children with CD, further prospective studies are required.

## Supplementary Material

The online Supplementary Material contains a table with additional information pertaining to the composition of enteral nutrition products used in the study.

## Figures and Tables

**Table 1:** Basic characteristics of the study population at the time of diagnosis.

Covariate	PEN group *N* = 42	Control group *N* = 45	*P* value^*∗*^
Age (yr), median [25–75%]	11.5 [9.2–13.7]	14.7 [12.7–16.3]	0.001
Sex, male, *n* (%)	30 (71)	31 (69)	0.82
Disease location at presentation, *n* (%)			
L1 (distal ileum ± cecum)	21 (50)	28 (62)	0.28
L2 (colon)	12 (28)	20 (44)	0.18
L3 (ileocolon)	13 (31)	9 (20)	0.32
L4a (upper GI, proximal to ligament of Treitz)	28 (67)	29 (64)	1.00
L4b (upper GI, distal to ligament of Treitz)	1 (2)	5 (11)	0.20
Perianal disease	4 (10)	7 (16)	1.00

yr, years. ^*∗*^Calculated with Chi-square or Fisher's exact test for categorical data and independent samples *t*-test for continuous variables.

**Table 2:** Remission rates and concomitant medication.

Covariate	PEN group	Control group	*P* value
Time between diagnosis and EEN initiation (mo), median (range)	2.0 (0–112)		
Route of EN administration, *n* (%)			
Oral	37/42 (88)		
Nasogastric	4/42 (10)		
PEG	1/42 (2)		
Follow-up time (mo), median [25–75%]	40 [25.5–79]	63 [52–87]	0.005^*∗*^
Length of remission (mo), median (range)	6.0 (0–36)	6.0 (range: 0–45)	0.96^*∗∗*^
Concomitant medication, *n* (%)			
Corticosteroids	26 (62)	35 (78)	0.16^*∗*^
Thiopurines	35 (83)	30 (67)	0.09^*∗*^
Anti-TNF agents	14 (33)	10 (22)	0.34^*∗*^
Methotrexate	7 (17)	2 (4)	0.08^*∗*^
Antibiotics	19 (45)	19 (42)	0.83^*∗*^
Time on PEN without concomitant medication (mo), median (range)^*∗∗*^	0 (0–36)		
0	23/43 (55%)		
1–6	13/42 (31%)		
7–12	2/42 (5%)		
13+	4/42 (9%)		
Length of remission with PEN alone, without concomitant medication (mo), median (range)^*∗∗*^	0 (0–16)		
0	25/42 (60%)		
1–6	14/42 (33%)		
7–12	2/42 (5%)		
13+	1/42 (2%)		

mo, months; EEN, exclusive enteral nutrition; PEN, partial enteral nutrition; EN, enteral nutrition; PEG, *Percutaneous endoscopic gastrostomy*; TNF, tumor necrosis factor. ^*∗*^Calculated with Chi-square or Fisher's exact test for categorical data and independent samples *t*-test for continuous variables. ^*∗∗*^Calculated with Mann–Whitney *U* tests.

**Table 3:** Laboratory parameters.

Covariate	PEN group	Control group	*P* value
Albumin (g/L), mean ± SD			
Before EEN/at diagnosis^*∗*^	35 ± 6	39 ± 6	0.008^*∗∗*^
After EEN/eight weeks after diagnosis^*∗*^	42 ± 5	42 ± 4	0.56^*∗∗*^
After three months of PEN/five months after diagnosis^*∗*^	39 ± 5	42 ± 3	0.007^*∗∗*^
CRP (mg/L), median (range)			
Before EEN/at diagnosis^*∗*^	21 (1–40)	10 (1–65)	0.03^*∗∗∗*^
After EEN/eight weeks after diagnosis^*∗*^	5 (0–107)	6.5 (1–48)	0.006^*∗∗∗*^
After three months of PEN/ five months after diagnosis^*∗*^	6.4 (0–180)	5 (1–84)	0.82^*∗∗∗*^

EEN, exclusive enteral nutrition; PEN, partial enteral nutrition; CRP, C-reactive protein. ^*∗*^Control group. ^*∗∗*^Calculated with Chi-square or Fisher's exact test for categorical data and independent samples *t*-test for continuous variables. ^*∗∗∗*^Calculated with Mann–Whitney *U* tests.

**Table 4:** Changes in weight, height, BMI, and wPCDAI over time.

Covariate	PEN group	Control group	*P* value
Weight (kg), mean ± SD			
Before EEN/at diagnosis^*∗*^	40.4 ± 13.0	51.5 ± 20.5	*P* = 0.002
After EEN/eight weeks after diagnosis^*∗*^	42.1 ± 13.2	52.2 ± 19.6	*P* = 0.005
After six months of PEN/eight months after diagnosis^*∗*^	45.1 ± 13.9	55.5 ± 21.3	*P* = 0.011
Height (m), mean ± SD			
Before EEN/at diagnosis^*∗*^	1.51 ± 0.17	1.58 ± 0.17	*P* = 0.061
After EEN/eight weeks after diagnosis^*∗*^	1.51 ± 0.17	1.57 ± 0.17	*P* = 0.12
After six months of PEN/eight months after diagnosis^*∗*^	1.53 ± 0.17	1.60 ± 0.17	*P* = 0.065
BMI, mean ± SD			
Before EEN/at diagnosis^*∗*^	16.9 ± 2.3	20.3 ± 4.9	0.001^*∗∗*^
After EEN/eight weeks after diagnosis^*∗*^	18.1 ± 4.2	20.4 ± 5.4	0.03^*∗∗*^
After six months of PEN/eight months after diagnosis^*∗*^	18.8 ± 2.9	21.3 ± 4.8	0.006^*∗∗*^
wPCDAI, median [25–75%]			
Before EEN/at diagnosis^*∗*^	30.0 [20–40]	30.0 [25–42.5]	0.70^*∗∗∗*^
After EEN/eight weeks after diagnosis^*∗*^	5.0 [0–12.5]	10.0 [7.5–17.5]	0.005^*∗∗∗*^
After three months of PEN/five months after diagnosis^*∗*^	7.5 [0–17.5]	11.3 [5–27.5]	0.16^*∗∗∗*^
After one year of PEN/one year after diagnosis^*∗*^	3.8 [0–13.8]	10.0 [0–20]	0.12^*∗∗∗*^
After two years of PEN/two years after diagnosis^*∗*^	17.5 [10–20]	10.0 [2.5–23.8]	0.43^*∗∗∗*^
After three years of PEN/three years after diagnosis^*∗*^	17.5 [5–17.5]	15.0 [0–21.9]	0.88^*∗∗∗*^

BMI, body mass index; EEN, exclusive enteral nutrition; PEN, partial enteral nutrition; wPCDAI, weighted Pediatric Crohn's Disease Activity Index. ^*∗*^Control group. ^*∗∗*^Calculated with Chi-square or Fisher's exact test for categorical data and independent samples *t*-test for continuous variables. ^*∗∗∗*^Calculated with Mann–Whitney *U* tests.
