# Supplementary material for: Maintenance of Remission with Partial Enteral Nutrition Therapy in Pediatric Crohn's Disease: A Retrospective Study
Source: Can J Gastroenterol Hepatol. 2017 May 8;2017:5873158. doi: 10.1155/2017/5873158 (PMC5439067; doi:10.1155/2017/5873158)
Supplement: Supplementary file 1 — The online Supplementary Material contains a table with additional information pertaining to the composition of enteral nutrition products used in the study. [file 5873158.f1.pdf]

Table S1. Composition of enteral nutrition products used.

Value (per 100ml)	Modulen IBD	Pediasure	Ensure
Energy (Kcal)	100	100	93
Protein (gr)	3.6	3	4.25
Carbohydrates (gr)	11	13.5	14.5
Fat (TOTAL) (gr)	4.7	3.8	2.53
MCT (gr)	1.2	0	0
Osmolarity (mOsm/l)	290		
Osmolality (mOsm/kg)	340	490	500
